# A New Statistical Method for Estimating Usual Intakes of Nearly-Daily Consumed Foods and Nutrients Through Use of Only One 24-hour Dietary Recall

**DOI:** 10.1093/jn/nxz070

**Published:** 2019-06-07

**Authors:** Hanqi Luo, Kevin W Dodd, Charles D Arnold, Reina Engle-Stone

**Affiliations:** 1 Department of Nutrition, University of California, Davis, CA; 2 National Cancer Institute, National Institutes of Health, Bethesda, MD

**Keywords:** dietary analysis, National Cancer Institute method, usual intake, statistical method, dietary recalls

## Abstract

**Background:**

To estimate usual intake distributions of dietary components, collection of nonconsecutive repeated 24-h dietary recalls is recommended, but resource limitations sometimes restrict data collection to single-day dietary data per person.

**Objectives:**

We developed a new statistical method, the NCI 1-d method, which uses single-day dietary data and an external within-person to between-person variance ratio to estimate population distributions of usual intake of nearly-daily consumed foods and nutrients.

**Methods:**

We used NHANES 2011–2014 data for men (*n* = 4938 and *n* = 4293 for the first and second 24-h recalls) to compare nutrient intake distributions of vitamin A, magnesium, folate, and vitamin E generated by the 1-d method (with use of only the first recall per person) with those from the NCI amount-only method (with use of all days of dietary intake per person). The within-person to between-person variance ratio from the amount-only model was used as the unbiased “external” estimate for the 1-d method. We also examined the effect of mis-specification of variance ratios on usual intake distributions.

**Results:**

The amount-only and 1-d methods estimated statistically equivalent median (25p, 75p): 647 (459, 890) compared with 648 (461, 886) μg retinol activity equivalents/d, 338 (268, 420) compared with 334 (266, 417) mg magnesium/d, 595 (458, 762) compared with 589 (456, 758) μg dietary folate equivalents/d, and 9.7 (7.3, 12.6) compared with 9.6 (7.3, 12.7) mg vitamin E/d. As the external variance ratios increased from 25% to 200% of the unbiased ratios, the prevalence of inadequate intake ranged from 53% to 43% for vitamin A, 57% to 55% for magnesium, 16% to 2% for folate, and 70% to 73% for vitamin E.

**Conclusions:**

The 1-d method is a viable statistical method for estimating usual intakes of nearly-daily consumed dietary components when the variance ratio is unbiased. Results are sensitive to variance ratio selection, so researchers should still collect replicate data where possible.

## Introduction

Dietary assessment provides important information for planning, monitoring, and evaluating nutrition intervention programs. For example, the World Health Organization recommends use of population nutrient intake distributions to select appropriate food fortification levels that minimize prevalence of inadequate nutrient intakes and avoid contributing to excessive intakes (1). Typically, these decisions require information on usual (that is, long-run average) dietary intake at the population level. However, usual intake is difficult to directly assess because of the considerable effort and resources involved in conducting frequent visits to households or developing and validating long-term assessment instruments such as Food Frequency Questionnaires. Thus, large dietary surveys typically adopt a hybrid approach of obtaining a small number of short-term dietary assessments, most commonly through use of 24-h dietary recall, and then statistical modeling (2) to estimate distributions of usual intake. The modeling treats overall variation in short-term intakes as a combination of between-person variation (that is, variation of usual dietary intake among individuals) and within-person variation (that is, day-to-day variation of dietary intake of an individual around that person’s usual intake). If repeated short-term assessments are collected in at least a subsample of the population, the 2 variance components can be estimated separately from the data, permitting adjustment for the effects of within-person variation.

Because of limitations in logistics and resources, some food consumption surveys and research studies have included only a single 24-h recall per person (3, 4). In these cases, the 2 variance components cannot be estimated separately with use of only the observed data. Researchers have addressed this problem by splitting the total variance in single-day data according to the within-person to between-person variance ratio calculated in a similar population from a different study, that is, an “external” estimate (5). Existing software programs such as PC-SIDE and IMAPP can use such external variance ratios to estimate distributions of usual intake from 1-d recall data (6, 7).

Over the years, the National Cancer Institute (NCI) method has been gaining popularity in the research world and is used to analyze data from research studies and large national surveys such as the National Health and Nutrition Examination Survey (NHANES) (8) and the Dutch National Food Consumption Survey (9). The NCI method provides flexibility in subgroup analysis and exploration of diet-disease relations (10). Researchers who enjoy the features of the NCI method have repeatedly expressed interest in applying it to single-day data. For example, the Micronutrient Intervention Modelling Project (MINIMOD) aimed to apply modelling approaches based on the NCI models (11) to national survey data from Ethiopia (3), where it was not logistically possible to collect multiple days of dietary intake data per respondent (12).

This paper describes a new statistical method: the NCI 1-d method. This method uses single-day dietary intake data and an external variance ratio to estimate population distributions of usual intakes of dietary components consumed by nearly everyone nearly every day (“nearly-daily”). The new method is implemented in a SAS macro designed to be compatible with the existing NCI macros, allowing the user to conduct the same kinds of analyses. In this paper, we use NHANES 2011–2014 data to compare the nutrient intake distributions generated by the 1-d method with use of only the first recall per person with those from the amount-only method, which used all days of dietary intake per person (13). Finally, because the selection of an appropriate external variance ratio is a critical component in the analysis of single-day data, we present sensitivity analyses that examine the effect of a mis-specified variance ratio on the distribution of usual intake and the prevalence of inadequate intake.

## Methods

### Common assumptions

We focus on the problem of estimating the distribution of usual intake for a single dietary component (14) that is consumed “nearly-daily” (that is, the intake of this dietary component for everyone on every day is zero for <5% of values), through use of what the NCI method documentation refers to as an “amount-only” model. We consider 24-h recalls assessing a single day’s intake as the available data, although the methods discussed apply equally well to other modalities such as diet records. Throughout, we assume that the 24-h recall is an unbiased instrument for measuring usual intake (15). This assumption does not imply that a 24-h recall measures an individual’s true intake on a particular day exactly, but rather that the mean of many such recalls approximates the individual’s usual intake. Other methods, such as those used in IMAPP, PC-SIDE, and the Multiple Source Method, make similar assumptions (16).

### Details of the amount-only method

The amount-only method requires that at least a subset of individuals have ≥2 repeated recalls, on nonconsecutive days (so that observations within an individual are independent). To account for the often-skewed distributions of single 24-h recall measurements, recalls are modeled after a Box-Cox transformation. Covariates may be included to represent the effect of personal characteristics, such as age, sex, or socioeconomic status on usual intake. Covariates may also be included to represent temporal effects such as day of the week. The model includes terms for within-person variation resulting from the individual’s day-to-day fluctuation around usual intake and other sources of random error, as well as a person-specific effect that allows an individual’s usual intake to vary from that predicted by individual-level covariates. The model can be written as:

$$\begin{array}{l} g(R_{ij};\lambda )=\beta_{0}+\sum_{k=1}^{K}\beta_{k}X_{ki}+\sum_{l=1}^{L}\beta_{l}X_{lij}+d_{ij}\\ \;d_{ij}\;=\;u_{i}+e_{ij} \end{array}$$ (1)

where *R_ij_* denotes the recall of individual *i* on day *j, g*(*x; λ*) = (*χ^λ^* − 1)/λ is the Box-Cox transformation, *X_ki_* is person *i*’s value of the *k*-th person-level covariate, *Z_lij_* is the value if the *l*-th temporal covariate for person *i* on day *j, β_k_* and *β_l_* are regression coefficients, and *d_ij_* is a zero-mean regression error that is further decomposed into a zero-mean person-specific effect *u_i_* and a zero-mean within-person error *e_ij_*. The transformation is assumed to produce normally distributed terms *u_i_* and *e_ij_*, which implies that *d_ij_* is also normally distributed. As mentioned previously, with ≥2 24-h recalls on a subset of individuals, it is possible to disaggregate the total residual variation (the variance of *d_ij_*) into between-person and within-person components (the variances of *u_i_* and *e_ij_*, respectively) (13).

**Figure 1:**
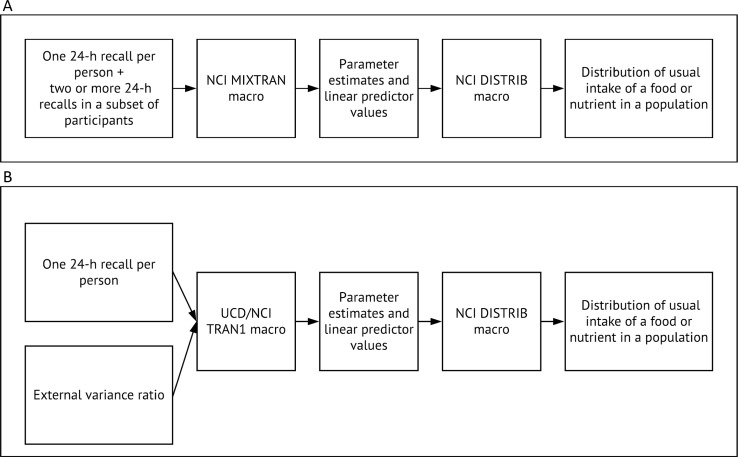
Illustration of the application of the NCI amount-only method (A) and NCI 1-d method (B) through use of SAS software. The external variance ratio refers to the ratio of within-person to between-person variation estimated from a different data set with replicate 24-h dietary recalls. NCI, National Cancer Institute; UCD, University of California, Davis.

The NCI method can be applied via a set of macros written in the SAS programming language, where 1 macro uses replicate dietary recalls to estimate model parameters (including the separate total variance into within-person and between-person variance components), and other macros use the output from the first to perform additional analyses. Two SAS macros, MIXTRAN and DISTRIB, are available to download from the NCI website (10). As illustrated in Figure 1A, users first apply MIXTRAN to intakes from 24-h recalls. MIXTRAN evaluates the effects of individual covariates on usual intake and generates parameter estimates and linear predictor values used as inputs for the DISTRIB macro. Then users apply DISTRIB with the parameters generated by MIXTRAN, which estimates the distribution of usual intake in a population (13) by analytically correcting for within-person variation.

### Details of the 1-d method

The model for the 1-d method is like that for the first part of the amount-only model, but with the *j* subscript suppressed because only 1 recall per person is available:

$$g(R_{i};\lambda )=\beta_{0}+\sum_{k=1}^{K}\beta_{k}X_{ki}+\sum_{l=1}^{L}\beta_{l}Z_{li}+d_{i}$$ (2)

Without 2 or more 24-h recalls on a subset of individuals, we can only estimate the variation in *d_i_*, and are unable to attribute variation to the between-person or within-person components. However, by applying an external variance ratio (for example, from another study), we can estimate the split of within-person and between-person variations within the total residual variation. Given variance of *d_i_* (that is, total variance) is known as V and ratio of within-person to between-person variance is *α*, between-person variance is calculated as $\frac{\text{V}}{1+\alpha }$ and within-person variance is $\frac{\text{V}\cdot \alpha }{1+\alpha }$ (Table 1). For example, if total variance of usual vitamin A intake is 20, and the ratio of within-person to between-person variance is 2, between-person variance is calculated as 20/(1 + 2) = 6.67 and within-person variance is 20 × 2/(1 + 2) = 13.3. Similarly, within-person and between-person variance can be calculated with use of different variance ratios as a starting point (Table 1).

**Table 1:** Formulas for calculation of between-person variance and within-person variance in nutrient intake through use of total variance (V) and different variance ratios

Variance ratio used to estimate between-person or within-person variance	Definition of variance ratio used to estimate between-person or within-person variance	Formula for calculation of between-person variance	Formula for calculation of within-person variance
Ratio of within-person to between-person variance (α)	$\frac{Within-person\;variance}{Between-person\;variance}$	$\frac{\text{V}}{1+\alpha }$	$\frac{\text{V}\cdot \alpha }{1+\alpha }$
Ratio of within-person to total variance (β)	$\frac{Within-person\;variance}{Total\;variance}$	V ⋅ (1 − β)	V ⋅ β
Ratio of between-person to total variance (γ)	$\frac{Between-person\;variance}{Total\;variance}$	V ⋅ γ	V ⋅ (1 − γ)

This approach requires users to identify an unbiased external variance ratio that is appropriate for the population being studied and the model being fit. In the general NCI model with covariates, between-person variation arises from “structured” variation in the individual-level covariates as well as “unstructured” variation in the person-specific random effects. Similarly, within-person variation arises from both structured variation because of temporal covariates and unstructured variation because of random within-person error. The required variance component ratio involves only terms corresponding to the unstructured types of variation. Ideally, the “external’” variance component ratio should be extracted from a model fit to replicate recalls collected on a representative sample from the same population, with use of the same set of covariates as is desired in the 1-d model. In practice, sensitivity analyses as described below can give a sense of how robust findings from the 1-d method are to different sources of bias in the external variance ratio.

Use of the 1-d method requires 2 SAS macros: TRAN1 and DISTRIB (Figure 1B). TRAN1 was designed by the NCI and University of California, Davis specifically for the 1-d method. The TRAN1 macro and a corresponding user manual are provided with this article (Supplemental Code 1 and Supplemental Manual 1) and example code and data are available online (Open Science Framework https://osf.io/aghdf/). TRAN1 first chooses an optimal Box-Cox transformation parameter for the selected nearly-daily consumed food or nutrient, then estimates regression coefficients and linear predictor values. Finally, it uses the external variance ratio (input by users) to estimate the between-person and within-person variance components. The output from TRAN1 is suitable for input to the DISTRIB macro, which is then used to estimate the distribution of usual intake as before.

### Procedural differences between the methods

While the 1-d model is the same as the amount-only model, MIXTRAN and TRAN1 take different approaches to estimating the required parameters. MIXTRAN jointly estimates the Box-Cox parameter and regression coefficients with use of the maximum likelihood technique, which produces an optimal transformation under the assumption of 2 independent normally distributed terms (*u_i_* and *e_ij_*). In contrast, TRAN1 tries to find an optimal transformation under the assumption of a single normally distributed term (*d_i_*). Briefly, the TRAN1 macro chooses the transformation yielding the most linear (as measured by *R*^2^) normal probability plot constructed from the first through 99th empirical percentiles of the residual distribution of *d_i_* when the Box-Cox parameter ranges from 0 (representing the natural log) to 1 in steps of size 0.01. This approach formalizes the commonly used graphical approach to check for normality, and easily extends to the complex survey situation (with survey-weighted percentiles), whereas other tests for normality such as the Kolmogorov-Smirnov and Anderson-Darling tests do not. Thus, the MIXTRAN and TRAN1 macros may choose different transformations, which would lead to different estimates of the regression parameters and variance components (partly because the parameters represent coefficients in a different scale, and partly because the 2 methods use different amounts of data). However, in practice, the qualitative results from regression models under different Box-Cox transformations tend to be similar unless the *λ* values are quite different. Also, if the *λ* values are the same, MIXTRAN and TRAN1 should provide similar estimates of regression coefficients, unless the relations between recalls and covariates differ systematically between the first recall and subsequent recalls in a data set.

### Data source and study population

To compare the amount-only and the 1-d methods, we combined data from up to 2 24-h recalls per person from the NHANES 2011–2012 (17) and 2013–2014 (18). The study population was subset to adult men aged ≥19 y (*n* = 4938 and *n* = 4293 for the first and second 24-h recalls). Because our aim was to compare the methods, rather than make inferences about the population, we selected adult men to avoid differences in nutrient requirements among subgroups of children and women as a result of growth, pregnancy, and lactation. We selected 4 nutrients for this comparison: vitamin A expressed as retinol activity equivalents (RAE), magnesium, folate expressed as dietary folate equivalents (DFE), and vitamin E. We selected these nutrients because the prevalence of inadequate intake was estimated to differ for each, and we anticipated that the distribution of intakes in relation to the estimated average requirement could affect the direction of bias resulting from mis-specification of the external variance component ratio. According to a previous NHANES analysis, the prevalence of inadequate intake was 55% for vitamin A (expressed as RAE), 55% for magnesium, 8% for folate (expressed as DFE), and 87% for vitamin E (19).

### Comparison of methods

We used SAS software (version 9.4) to conduct all analyses. The amount-only model was fit through use of all the recall data with the MIXTRAN macro, then percentiles of usual nutrient intake and the prevalence of inadequate intake were obtained from the DISTRIB macro with the MIXTRAN parameter estimates. We also calculated the ratio of the within-person to between-person variance components from the amount-only model to be used as the external variance ratio in the 1-d method. The 1-d model was fit with use of only the first recall per person with the TRAN1 macro, with the external variance ratio from the corresponding MIXTRAN run. Both the MIXTRAN and TRAN1 models included person-level covariates: age (as a continuous variable) and indicator variables for race/ethnicity category. Both models also included an indicator for whether the recall referred to a weekend (Friday–Sunday) compared with a weekday (Monday–Thursday) as the only temporal covariate. These covariates were chosen because *1*) dietary recommendations differ by age, *2*) because racial/ethnic differences in usual intakes are often of interest, and *3*) because historical data suggest that daily intakes may differ by weekend/weekday status. In addition, this set of covariates exemplifies the flexibility of the NCI modeling approach. Then percentiles of usual intake and prevalence of inadequate intake were obtained from the DISTRIB macro with use of TRAN1 parameter estimates. For later comparisons, we extracted the estimates of transformation parameters, variance components (and their sum, representing total residual variance), selected percentiles of the estimated usual intake distributions, and prevalence of inadequate intake from the 2 methods for each nutrient.

Standard errors of estimates were calculated through use of the balanced repeated replication technique with 32 sets of replicate weights (8). We used equivalence testing with equivalence margins of 5% of the amount-only estimate (for means and percentiles), and 0.5 percentage points (for prevalence estimates) to evaluate whether means, percentiles, and prevalence of inadequate intake estimated from the 2 methods were equivalent (20).

### Sensitivity analysis

The amount-only and 1-d methods were applied to the same data set, representing the same population, and with use of the same covariates; hence, the variance ratio estimated by the amount-only method is assumed to be an unbiased variance ratio for the 1-d method in this analysis. In practice, researchers analyzing data sets with only single-day data will need to use an external variance ratio from a similar, but not the same, study population, and the external variance ratio might be biased. To evaluate the difference in usual nutrient distribution resulting from the use of different external variance ratios, we repeated the analyses described above and applied a range of variance ratios (25%, 50%, 75%, 90%, 110%, 125%, 150%, 175%, and 200% of the unbiased variance ratio for each nutrient).

## Results

### Comparison of methods

Table 2 shows that the amount-only method and the 1-d method selected different transformations; thus, the total residual variance differed between the 2 methods for all 4 nutrients (vitamin A: 15.7 compared with 20.7; magnesium: 6.3 compared with 1.0; folate: 6.7 compared with 2.4; vitamin E: 1.1 compared with 0.7). As a consequence, the within-person and between-person variance components from the 2 methods were also substantially different, even though the variance ratio was held constant between methods for each nutrient. For example, for vitamin A intake analyzed through use of the amount-only method, the within-person variance was 10.450 and the between-person variance was 5.220, whereas the corresponding values from the 1-d method were 13.829 and 6.909. Similarly, for magnesium, the within-person and between-person variances were both ~6 times greater than those estimated by the 1-d method. Despite this, the amount-only and 1-d methods estimated functionally identical means, 25th, 50th, and 75th percentiles, and prevalence of inadequate intake for all nutrients examined (Table 3). Median (p25, p75) intake was 647 (459, 890) compared with 648 (461, 886) μg RAE/d, 338 (268, 420) compared with 334 (266, 417) mg magnesium/d, 595 (458, 762) compared with 589 (456, 758) μg DFE/d, and 9.7 (7.3, 12.6) compared with 9.6 (7.3, 12.7) mg vitamin E/d through use of the amount-only and 1-d methods, respectively. An example of the overlap of the entire intake distribution estimated by the amount-only and 1-d methods is shown in **Supplemental Figure 1**. This result confirmed the initial expectation that the characteristics of the estimated usual intake distributions are fairly robust to the choice of Box-Cox parameter, so long as the variance component ratio is appropriate.

**Table 2:** Box-Cox transformation *λ* and variances and vitamin A, magnesium, folate, and vitamin E intakes estimated by the NCI amount only and NCI 1-d methods among adult men in NHANES 2011–2014^1^

Parameters	Vitamin A, μg RAE/d		Magnesium, mg/d		Folate, μg DFE/d		Vitamin E, mg/d	
	NCI amount-only	NCI 1-d	NCI amount-only	NCI 1-d	NCI amount-only	NCI 1-d	NCI amount-only	NCI 1-d
*λ* of the transformation	0.237	0.260	0.284	0.130	0.219	0.140	0.203	0.110
Total variance	15.670	20.738	6.306	1.022	6.654	2.381	1.144	0.758
Ratio of within-person to between-person variance	2.002	2.002	1.072	1.072	1.820	1.820	1.723	1.723
Within-person variance	10.450	13.829	3.262	0.529	4.294	1.536	0.724	0.480
Between-person variance	5.220	6.909	3.044	0.493	2.360	0.844	0.420	0.279

1 The ratio of within-person and between-person variance was calculated with use of the NCI amount-only method and used as an input in the NCI 1-d method. *n*_1_ = 4938 and *n*_2_ = 4293 for the first and second 24-h recalls for the amount-only method and *n* = 4938 for the 1-d method. DFE, dietary folate equivalents; NCI, National Cancer Institute; NHANES, National Health and Nutrition Examination Survey; RAE, retinol activity equivalents.

**Table 3:** Mean, prevalence of inadequate intake, and 25th, 50th, and 75th percentiles of usual vitamin A, magnesium, folate, and vitamin E intake among adult men in the United States as estimated by the NCI amount-only method and NCI 1-d method with NHANES 2011–2014 data^1^

Parameters	Vitamin A, μg RAE/d		Magnesium, mg/d		Folate, μg DFE/d		Vitamin E, mg/d	
	NCI amount-only	NCI 1-d	NCI amount-only	NCI 1-d	NCI amount-only	NCI 1-d	NCI amount-only	NCI 1-d
Mean	708.0 (19.2)	704.6 (21.8)	351.3 (3.3)	350.7 (3.5)	628.7 (6.4)	626.6 (6.4)	10.3 (0.1)	10.4 (0.1)
25th percentile	459.3 (11.7)	460.6 (9.5)	267.7 (2.6)	266.4 (2.2)	458.2 (7.6)	455.5 (4.8)	7.3 (0.1)	7.3 (0.1)
50th percentile	647.4 (14.2)	647.5 (17.3)	337.6 (3.0)	334.3 (2.9)	594.9 (6.6)	589.4 (6.1)	9.7 (0.1)	9.6 (0.1)
75th percentile	890.0 (25.0)	885.7 (29.6)	420.0 (4.4)	417.2 (4.3)	762.4 (7.9)	757.5 (7.8)	12.6 (0.2)	12.7 (0.2)
Inadequate intake, %	47.1 (1.8)	47.1 (2.1)	54.3 (1.1)	55.5 (1.1)	6.1 (0.8)	6.0 (0.4)	70.6 (1.2)	70.6 (1.2)

1 Values in parentheses are standard errors. *n*_1_ = 4938 and *n*_2_ = 4293 for the first and second 24-h recalls for the amount-only method and *n* = 4938 for the 1-d method. The ratio of within-person and between-person variance was calculated through use of the NCI amount-only method and used as an input in the NCI 1-d method. For all parameters, the estimates via each method are functionally identical for applied use as well as statistically significant by equivalence testing (*P* < 0.05). DFE, dietary folate equivalents; NCI, National Cancer Institute; NHANES, National Health and Nutrition Examination Survey; RAE, retinol activity equivalents.

### Sensitivity analysis

**Figure 2:**
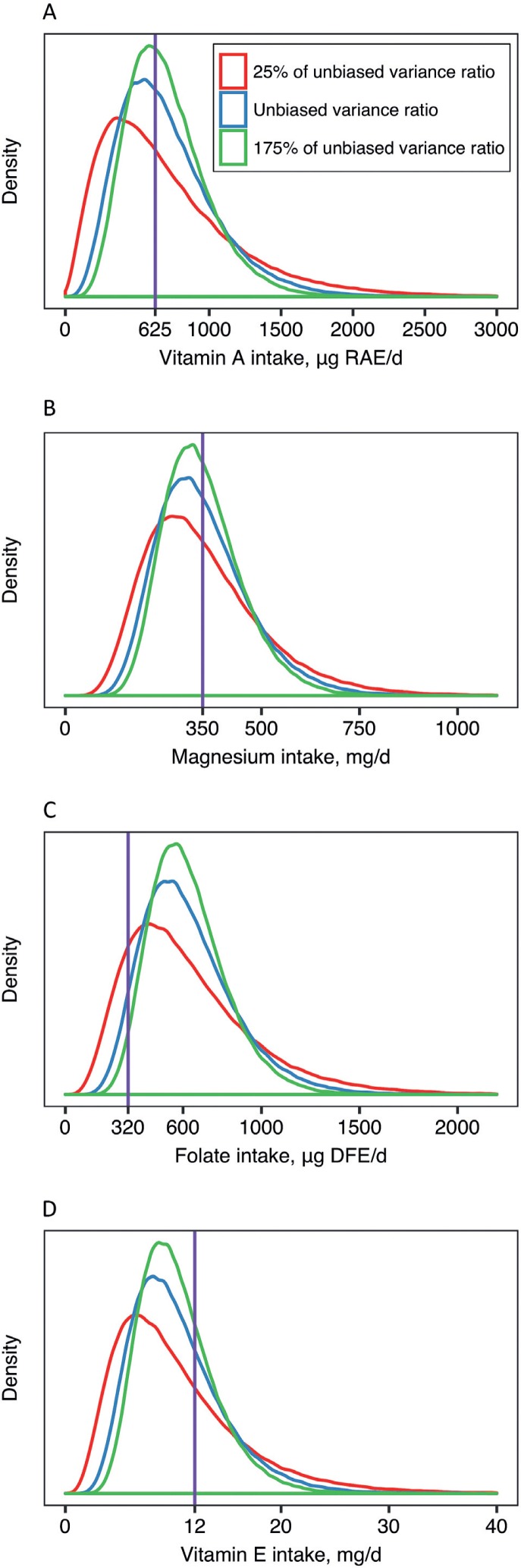
Distributions of usual vitamin A intake (μg RAE/d) (A), magnesium intake (mg/d) (B), folate intake (μg DFE/d) (C), and vitamin E intake (mg/d) (D) among adult men in the United States estimated by the National Cancer Institute 1-d method with use of different inputs for the assumed within-person to between-person variance ratio. The vertical purple line indicates the estimated average requirement for intake of each nutrient among adult men. DFE, dietary folate equivalents; RAE, retinol activity equivalents.

**Figure 3:**
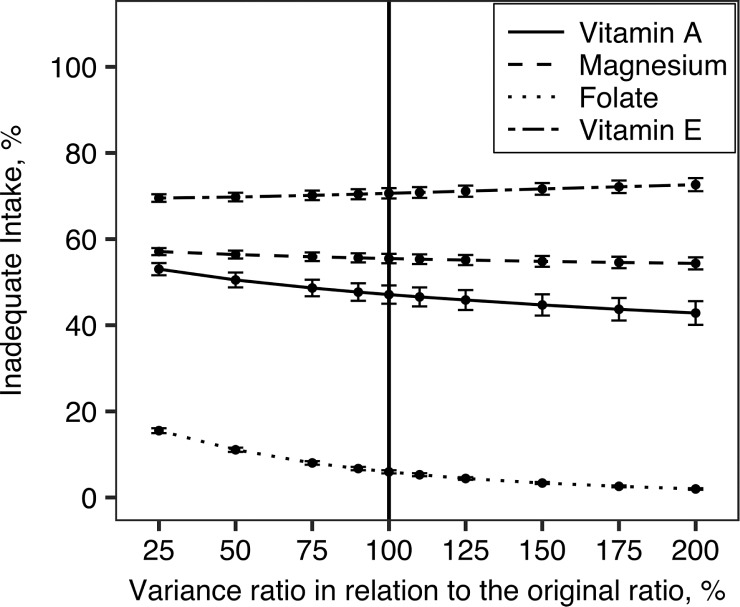
Estimated prevalence of inadequate intake of vitamin A, magnesium, folate, and vitamin E among adult men in the United States estimated by the NCI 1-d method with use of different inputs for the assumed within-person to between-person variance ratios. Error bars represent the standard error of the prevalence of inadequate intake. The solid vertical line represents the scenario in which the unbiased within-to-between variance ratio was used (that is, the ratio calculated from the same data set through use of the NCI amount-only method and 2 d of recalls). NCI, National Cancer Institute.

For the 1-d method, the estimated mean usual intake was unaffected by mis-specifying the variance ratio, because of the way the amount-only (and hence the 1-d) methods adjust for within-person variation in the DISTRIB macro. However, underestimating the variance ratio produced estimated usual intake distributions that were flatter and more spread out than those produced by the unbiased ratio, while overestimation produced the opposite effect— distributions that were taller and with less spread (Figure 2). In either case, biased variance ratios caused biased distributions, medians, and percentiles of usual nutrient intakes. Biased variance ratios also affected the estimated prevalence of inadequate nutrient intake (Figure 3). As the variance ratios increased from 25% to 200% of the unbiased variance ratios, the estimated prevalence of inadequate intake dropped from 53% to 43% for vitamin A, 57% to 55% for magnesium, and 16% to 2% for folate, but the estimated prevalence of inadequate vitamin E intake increased from 70% to 73%.

## Discussion

The 1-d method is a viable statistical method for estimating usual intakes of nearly-daily consumed dietary components with use of only 1 24-h dietary recall, if the selected external variance ratio is unbiased. In this case, the 1-d method estimated the distributions, percentiles, and means of usual intakes and prevalence of inadequate intake equivalently (in fact, almost identically) to the NCI amount-only method. Even though a biased variance ratio does not affect the means of usual nutrient intakes, it can change the distributions and percentiles of usual nutrient intakes and prevalence of inadequate intake.

The 1-d method has several advantages. First, it shares the ability of the amount-only method to incorporate the effect of covariates that influence nutrient intakes. Second, the 1-d method was developed to be publicly available and easy-to-use for a broad research community. The TRAN1 macro and user guide are available in the supplemental materials for this paper and the sample data and code are on the Open Science Framework website. Users have the flexibility of modifying the sample code to incorporate covariates and generate results by subgroups for their own dietary studies. Moreover, because the TRAN1 macro provides a drop-in replacement for the MIXTRAN macro, it can be used in conjunction with the NCI macro INDIVINT to provide error-corrected estimates of regression slopes in health outcome models where usual intake is considered a continuous exposure (21). Thus, the development and promotion of the 1-d method can be a particularly useful addition to the existing dietary analysis methods.

When applying the 1-d method, users should also be aware of the following points. As shown in Table 2, the amount-only and the 1-d methods selected different transformations when fitting the model, leading to different estimated values of total, within-person, and between-person variances. However, the striking similarity between the final results indicates that the ratio of variance components is the critical parameter, not the components themselves (Table 3). Preliminary results from a literature review suggest that there is wide variation in the variance component ratios presented in the literature (22). It follows that users should be careful when applying an external variance ratio from studies of a different population or collected at a different time compared to their own study population. In addition, when covariates are included in the model, the external variance component ratio should be based on estimates from a comparable model with similar covariates. Failure to do so will induce bias into the variance ratio. Our finding that a biased variance ratio can affect percentiles and prevalence of inadequacy of usual nutrient intakes is consistent with previous research (5, 22, 23). In this case, sensitivity analyses are necessary. After applying the 1-d method with the selected external variance ratio, users should also use a range of variance ratios that are both smaller and bigger than the selected variance ratio to see how the parameters of interest change. If the parameters of interest are relatively stable regardless of the change in variance ratio, users can assume that the estimated usual intake distribution is robust; however, if the parameters of interest change substantially corresponding to a small change in variance ratio, users should interpret their results with caution. Furthermore, we defined the variance ratio as the ratio of within-person to between-person variances in this paper. Other published studies might present the variance ratio as the ratio of within-person to total variances, or the ratio of between-person to total variances. The TRAN1 macro allows users to specify which type of variance ratio is desired. When extracting the external variance ratio from a research study, users should scrutinize the analysis method of that research study and identify the type of variance ratio used, which is not always clear (22).

The 1-d method, as other 1-d methods to estimate usual nutrient distribution, assumes that the first day of dietary recalls used is not systematically different from the second day (and the rest of) dietary recalls. In the NCI amount-only method, the second day dietary recall also contributes to estimating the effect of covariates. If there is a systematic difference between 2 d of dietary recalls, the 1-d method could estimate different coefficients of covariates compared to the amount-only method, which can lead to estimating a biased usual nutrient distribution with single-day data. Furthermore, in this paper, we only analyzed vitamin A, magnesium, folate, and vitamin E intakes in adult men in the United States. The comparisons of the amount-only and the 1-d methods are likely to be similar for other population groups, such as women and children, and nutrients, but the effect of the external variance ratio may differ. Finally, we have focused only on developing the 1-d method that is applied to nearly-daily consumed dietary components; a similar approach for episodically consumed foods and nutrients will be developed in the future.

In conclusion, the new 1-d method can be used to estimate population usual intakes of nearly-daily consumed foods and nutrients for surveys or research studies that lack repeated dietary recalls. The selection of an external variance ratio is a critical step in the use of this method. Little guidance exists regarding what to assume if this value is not available, so we recommend that users conduct sensitivity analyses to assess the robustness of their conclusions. This method is intended as a tool to better use existing data sets that include only 1 d of dietary intake data per person. We strongly recommend that researchers still collect repeated dietary recalls on nonconsecutive days where feasible.

## References

[1] World Health Organization Guidelines on food fortification with micronutrients [Internet]. 2006; Geneva: World Health Organization Available from: http://www.who.int/nutrition/publications/micronutrients/9241594012/en/.

[2] Nusser SM, Carriquiry AL, Dodd KW, Fuller WA A semiparametric transformation approach to estimating usual daily intake distributions. J Am Statist Assoc 2012;91:1440–9.

[3] Ethiopian Public Health Institute Ethiopia National Food Consumption Survey. Addis Ababa (Ethiopia): Ethiopian Public Health Institute (EPHI); 2013.

[4] Rivera JA, Pedraza LS, Aburto TC, Batis C, Sánchez-Pimienta TG, González de Cosío T, López-Olmedo N, Pedroza-Tobías A Overview of the dietary intakes of the Mexican population: results from the National Health and Nutrition Survey 2012. J Nutr 2016;146:1851S–5S. 27511939 10.3945/jn.115.221275

[5] Jahns L, Arab L, Carriquiry A, Popkin BM The use of external within-person variance estimates to adjust nutrient intake distributions over time and across populations. Public Health Nutr 2005;8:69–76. 15705247 10.1079/phn2005671

[6] Iowa State University PC-Side [Internet]. 1st ed. Ames (IA): Iowa State University Available from: side.stat.iastate.edu/pc-side.php.

[7] Iowa State University IMAPP [Internet]. 1st ed. Ames, Iowa: Iowa State University Available from: side.stat.iastate.edu/imapp.php.

[8] Centers for Disease Control and Prevention (CDC) NHANES—NHANES III Web Tutorial—Variance Estimation [Internet]. cdc.gov. [cited 2018 Sep 12]. Available from: https://www.cdc.gov/nchs/tutorials/NHANES/SurveyDesign/VarianceEstimation/intro_iii.htm.

[9] Verkaik-Kloosterman J, Dodd KW, Dekkers ALM, van ’t Veer P, Ocké MC A three-part, mixed-effects model to estimate the habitual total vitamin D intake distribution from food and dietary supplements in Dutch young children. J Nutr 2011;141:2055–63. 21956963 10.3945/jn.111.142398

[10] National Cancer Institute Usual dietary intakes: the NCI method [Internet]. epi.grants.cancer.gov. [cited 2018 Sep 12]. Available from: https://epi.grants.cancer.gov/diet/usualintakes/method.html.

[11] Engle-Stone R, Nankap M, Ndjebayi AO, Vosti SA, Brown KH Estimating the effective coverage of programs to control vitamin A deficiency and its consequences among women and young children in Cameroon. Food Nutr Bull 2015;36:S149–71. 26385984 10.1177/0379572115595888

[12] Moges T, Tesfaye B, Kebebe T, French C, Asfar E, Kagin J, Luo H, Engle-Stone R, Vosti SA Predicted impacts of fortifying edible oil with vitamin A on the prevalence of inadequate intake and estimated lives saved of children in Ethiopia. The 4th Health Science Congress Addis Ababa (Ethiopia); May, 2019.

[13] Tooze JA, Kipnis V, Buckman DW, Carroll RJ, Freedman LS, Guenther PM, Krebs-Smith SM, Subar AF, Dodd KW A mixed-effects model approach for estimating the distribution of usual intake of nutrients: the NCI method. Stat Med 2010;29:2857–68. 20862656 10.1002/sim.4063 PMC3865776

[14] Dodd KW, Guenther PM, Freedman LS, Subar AF, Kipnis V, Midthune D, Tooze JA, Krebs-Smith SM Statistical methods for estimating usual intake of nutrients and foods: a review of the theory. J Am Diet Assoc 2006;106:1640–50. 17000197 10.1016/j.jada.2006.07.011

[15] Tooze JA, Midthune D, Dodd KW, Freedman LS, Krebs-Smith SM, Subar AF, Guenther PM, Carroll RJ, Kipnis V A new statistical method for estimating the usual intake of episodically consumed foods with application to their distribution. J Am Diet Assoc 2006;106:1575–87. 17000190 10.1016/j.jada.2006.07.003 PMC2517157

[16] Laureano G, Torman V, Crispim S, Dekkers A, Camey S Comparison of the ISU, NCI, MSM, and SPADE methods for estimating usual intake: a simulation study of nutrients consumed daily. Nutrients 2016;8:166–11. 26999193 10.3390/nu8030166 PMC4808894

[17] Centers for Disease Control and Prevention (CDC) National Health and Nutrition Examination Survey 2011–2012 [Internet]. www.cdc.gov. [cited 2018 Sep 12]. Available from: https://www.cdc.gov/nchs/nhanes/Search/DataPage.aspx?Component=Questionnaire&CycleBeginYear=2011.

[18] Centers for Disease Control and Prevention (CDC) National Health and Nutrition Examination Survey 2013–2014 [Internet]. www.cdc.gov. [cited 2018 Sep 12]. Available from: https://www.cdc.gov/nchs/nhanes/continuousnhanes/default.aspx?BeginYear=2013.

[19] Blumberg JB, Frei B, Fulgoni VL, Weaver CM, Zeisel SH Contribution of dietary supplements to nutritional adequacy in various adult age groups. Nutrients 2017;9:1325. 10.3390/nu9121325 PMC5748775 29211007

[20] Walker E, Nowacki AS Understanding equivalence and noninferiority testing. J Gen Intern Med 2010;26:192–6. 20857339 10.1007/s11606-010-1513-8 PMC3019319

[21] Kipnis V, Midthune D, Buckman DW, Dodd KW, Guenther PM, Krebs-Smith SM, Subar AF, Tooze JA, Carroll RJ, Freedman LS Modeling data with excess zeros and measurement error: application to evaluating relationships between episodically consumed foods and health outcomes. Biometrics 2009;65:1003–10. 19302405 10.1111/j.1541-0420.2009.01223.x PMC2881223

[22] French C, Arsenault JE, Arnold C, Luo H, Engle-Stone R Application of external variance estimates for modeling usual nutrient intake distributions: literature review and simulation analysis. Nutrition 2018 Boston, MA; 2018.

[23] Jahns L, Carriquiry A, Arab L, Mroz TA, Popkin BM Within- and between-person variation in nutrient intakes of Russian and U.S. children differs by sex and age. J Nutr 2004;134:3114–20. 15514284 10.1093/jn/134.11.3114

