# Supplementary material for: A New Statistical Method for Estimating Usual Intakes of Nearly-Daily Consumed Foods and Nutrients Through Use of Only One 24-hour Dietary Recall
Source: J Nutr. 2019 Jun 7;149(9):1667–73. doi: 10.1093/jn/nxz070 (PMC6862942; doi:10.1093/jn/nxz070)
Supplement: Supplementary file 1 [file JN-2019-JN-NXZ070-S1.pdf]

Supplementary data

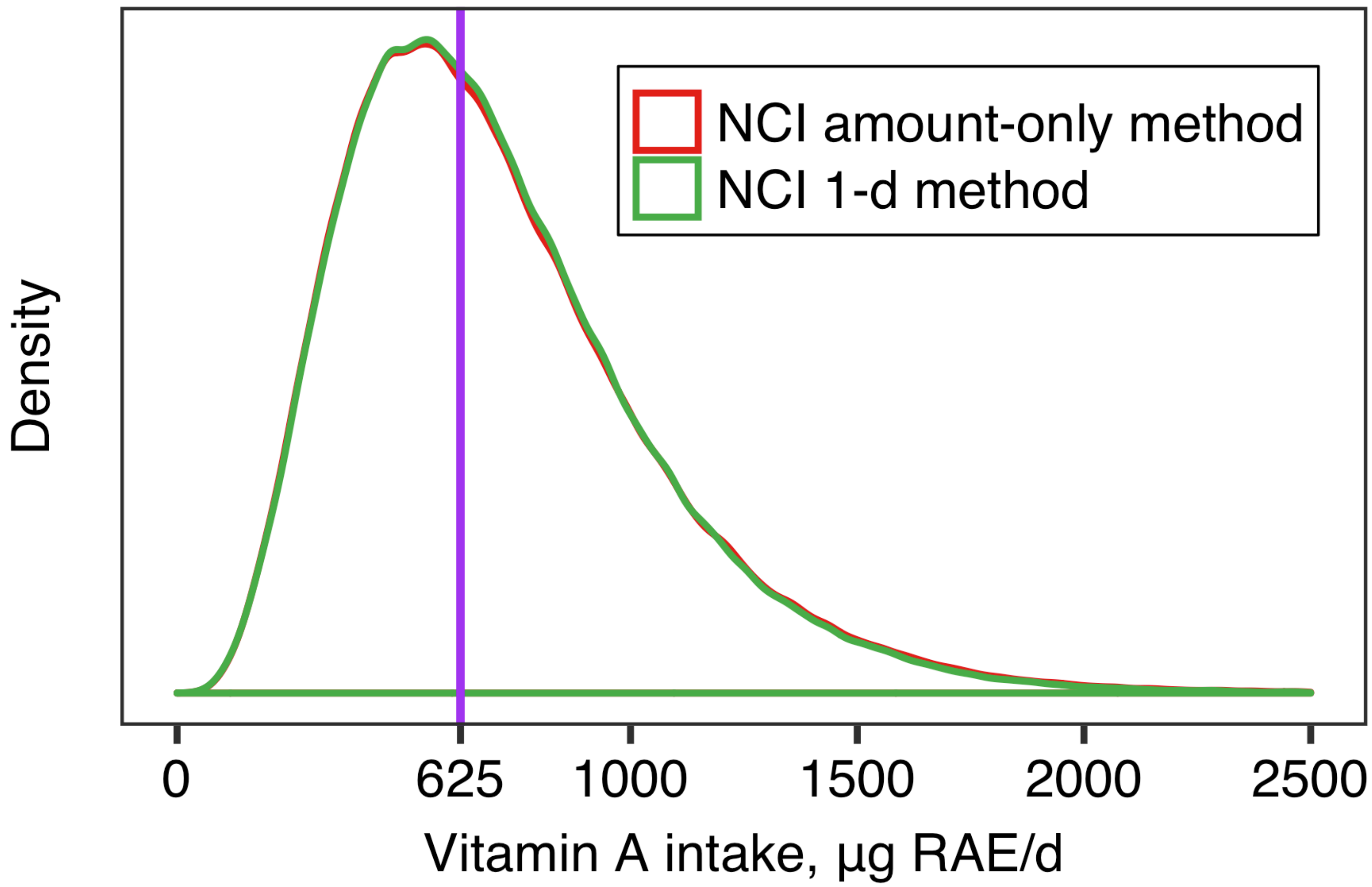

Supplemental figure 1: Distribution of usual vitamin A intake (µg RAE/d) among U.S. adult males estimated by the NCI amount-only method and the NCI 1-d method[1]. The purple line indicates the Estimated Average Requirement of vitamin A intake (µg/d). RAE, retinol activity equivalents.
