# Supplementary material for: A New Statistical Method for Estimating Usual Intakes of Nearly-Daily Consumed Foods and Nutrients Through Use of Only One 24-hour Dietary Recall
Source: J Nutr. 2019 Jun 7;149(9):1667–73. doi: 10.1093/jn/nxz070 (PMC6862942; doi:10.1093/jn/nxz070)
Supplement: Supplementary file 2 [file JN-2019-JN-NXZ070-S2.pdf]

Supplementary data

Supplemental Macro 1

```
/******************************************************************************/
/**********************TRAN1 MACRO********************************************/
/*Objective: The TRAN1 macro is designed for dietary surveys that only have one dietary recall. It is used for
the analysis of foods and nutrients consumed every day. The output datasets from the TRAN1 macro are
used by the DISTRIB macro to estimate the distribution of usual intake.
The syntax for calling the macro is:

%macro tran1
(data=, subject =, response=, covars =, ratio = , ratioType =, weight=,  weekend = , foodtype =, outlib = );

Inputs:
"data" Specifies the dataset to be used.
"subject" Specifies the variable that uniquely identifies each subject.
ìresponse" Specifies the variable name of food or nutrient in the 24-hr recall.
"covars" Specifies a list of covariates (NOT including weekend variable). Covariates must be separated by
spaces. Covariates can be either continuous variables and/or binary variables. For categorical variables,
users need to recode categorical variables into multiple binary variables. This input is optional.
ìRatioî Specifies the ratio of variance component (can be in either decimal or fraction forms)
ìratioTypeî Specifies the type of variance ratio. The possible values are
WIVtoTotal: Within-person to total variance.
BIVtoTotal: Between-person to total variance.
WIVtoBIV: Within to Between-person variance
"weight" Specifies the survey weight. The input is optional. If weight is not specified, all the subjects will be
assumed having equal weights.  In general, using the NCI macro, the survey weight should be integer. In
tran1, because we just use part of the NCI macro, you could have survey weight as non-integer.
"weekend" Specifies the weekend (Fri.-Sun.) indicator variable to account for a weekend effect.  A value of
1 represents a Fri.-Sun. record, and a value of 0 represents a Mon.-Thurs. record.
ìfoodtypeî Specifies a name for the analysis, used to identify the output data sets.  This value can be the
same as the response variable.
"outlib" Specifies a directory where output data sets are stored.  If outlib is not specified, the output
datasets will be stored under work folder in SAS.  This input is optional.

data set names 'minivalue', '_Mse', '_Params', '_Params2', '_pred', '_pred2', '_reg', '_data', '_results',
'_Pctile', '_Pctilet', '_Rsquarestatistics' are reserved for this macro.

*/



%macro tran1(data=, response=, covars =, ratio = , ratioType =,
 weight=,  weekend = , subject = , foodtype =, outlib = );

/*************************/
/* set up loop parameters*/
/*************************/
%let steplam = .01;
%let first_step = 0;
%let last_step = 100; * will change later;

/**************************************************/
/* check if the reserved dataset names are avaiable*/
/**************************************************/
```

Supplementary data

```
%if %sysfunc(exist(minivalue))| %sysfunc(exist(_Mse)) | %sysfunc(exist(_Params)) |
   %sysfunc(exist(_Params2)) | %sysfunc(exist(_pred)) | %sysfunc(exist(_pred2)) |
           %sysfunc(exist(_reg)) | %sysfunc(exist(_data)) | %sysfunc(exist(_results)) |
   %sysfunc(exist(_Pctile)) | %sysfunc(exist(_Pctilet)) | %sysfunc(exist(_Rsquarestatistics))
%then %do;
     %put
'**************************************************************************************'
;
     %put "** Warning **" ;
     %put "** data set names 'minivalue', '_Mse', '_Params', '_Params2', '_pred', '_pred2', '_reg', '_data',
'_results', '_Pctile', '_Pctilet', '_Rsquarestatistics' are reserved for this macro.";
     %put "** These datasets will be deleted.";
     %put
'**************************************************************************************'
;
%end;

/* delete useless dataset*/
proc delete data = minvalue
                _Mse
                _Params
                _Params2
                _pred
                _pred2
                _reg
                                        _data
                                        _results
                                        _pctile
                                        _pctilet
                _Rsquarestatistics
                _weight(gennum=all)
                _data;
run;

quit;


/***********************************/
/* check if the inputs are correct */
/***********************************/
* if there is no input for food type, put it the same as response **;
%if &foodtype. eq %str() %then &foodtype = &response;



%if &data eq %str() %then %do;
     %put '*********************************************************';
     %put "** ERROR **" ;
     %put "** No input data";
     %put "** Processing of the macro TRAN1 will be stopped.";
     %put '*********************************************************';
     %return;
%end;
```

Supplementary data

```
%if &response eq %str() %then %do;
    %put '**********************************************************';
    %put "** ERROR **" ;
    %put "** No nutrients or food";
    %put "** Processing of the macro TRAN1 will be stopped.";
    %put '**********************************************************';
    %return;
%end;

%if &weekend eq %str() %then %do;
    %put '**********************************************************';
    %put "** ERROR **" ;
    %put "** You need to specify 'weekend': if the survey date is a weekend";
    %put "** Processing of the macro TRAN1 will be stopped.";
    %put '**********************************************************';
    %return;
%end;

%if &subject eq %str() %then %do;
    %put '**********************************************************';
    %put "** ERROR **" ;
    %put "** You need to specify 'subject': the unique IDs of participants";
    %put "** Processing of the macro TRAN1 will be stopped.";
    %put '**********************************************************';
    %return;
%end;

%if &outlib eq %str() %then %do;
    %put '**********************************************************';
    %put "** WANRING **" ;
    %put "** No outlib indicated. Data will be saved at work folder";
    %put "** Processing of the macro TRAN1 will be stopped.";
    %put '**********************************************************';
    %let &outlib = work;
    %return;
%end;

%let RatioType = %upcase(&RatioType);
%if &RatioType eq %str(WIVTOTOTAL) & (&ratio < 0| &ratio > 1)  %then %do;
    %put '**********************************************************';
    %put "** ERROR **" ;
    %put "** &ratioType is between 0 and 1";
    %put "** Processing of the macro TRAN1 will be stopped.";
    %put '**********************************************************';
    %let &outlib = work;
    %return;
%end;

%if &RatioType eq %str(BIVTOTOTAL) & (&ratio < 0| &ratio > 1)  %then %do;
    %put '**********************************************************';
    %put "** ERROR **" ;
    %put "** &ratioType is between 0 and 1";
    %put "** Processing of the macro TRAN1 will be stopped.";
    %put '**********************************************************';
```

```
     %let &outlib = work;
     %return;
%end;




/*********************************************/
/* combine "weekend" and covars as covariates*/
/*********************************************/
%let covars =  &covars &weekend;

/* the weight variable  */
%if &weight eq %str() %then %do ;       /* if no replicate variable */
  *%put no weight variable;
  %let weight = %str(dummywt);       /* assign dummy weight if user  did not supply a surveyweight variable.
A value of 1 will be supplied */
  *%put ## weight variable is &weight;
%end; /* no replicate variable */
%else %do ;                              /* replicate variable supplied */
  *%put ## weight variable is &weight;
%end;  /* of replicate variable supplied */

/*put weight command for proc reg*/
%let weight_command = weight &weight; /* for the proc reg */
*%put &weight_command;


/******************************************************************************/
/** variance ratio **********************************************************/
/** Calculate WIV to Total variance ratio based on the ratio and ratio type*/
/*****************************************************************************/

%if  &RatioType eq %str(WIVTOTOTAL) %then %let vratio = &ratio;
   %else %if  &RatioType eq %str(WIVTOBIV) %then %let vratio = &ratio / (1 + &ratio);
   %else %if  &RatioType eq %str(BIVTOTotal) %then %let vratio = 1 - &ratio;
   %else %do;
                  %put '************************************************************';
            %put ## Error: the user must provide Ratio type;
                                    %put ## your ratio type is &ratioType;
            %put ## TRAN1 will not execute properly;
                                    %put
'************************************************************';
   %return;
%end;
run;

/*********************************************************************************/
/*---make datasets------------------------------------------------------------------*/
/*********************************************************************************/
data _data;
  set &data;
  if &response = . then delete; *remove values without the a nutrient value *;
  %If &weight ne %str(dummywt) %then %do;
  /*
```

Supplementary data

```
   If &weight ne int(&weight) then do ;
     put;
     put '**********************************************************';
     put "** ERROR **" ;
     put "**  The weight variable &weight is not an integer.";
     put "**   Processing of the macro TRAN1 will be stopped.";
     put '**********************************************************';
     put;
     stop;
   end;             ** of replicate_var ne int(replicate_var);
 */
 %end;              /* of if replicate variable supplied */

   %else %do ;          /* assign a dummy weight variable, value =1 */
   dummywt=1;
           %put no weight variable;
 %end;
 run;


/* sort the data based on the seqn*/
proc sort data = _data; by &subject; run;


/* check if the output dataset */

%if %eval(%sysfunc(sum(%length(&foodtype.),%length(%str(Params_)))) > 32) %then %do;
     %put '**********************************************************';
     %put "** ERROR **" ;
     %put "** The variable name of nutrient/Food (&foodtype.) is too long";
     %put "** The user need to shorten the variable name";
     %put "** Processing of the macro TRAN1 will be stopped.";
     %put '**********************************************************';
     %return;
%end;


/* check if the output dataset */
%if %eval(%sysfunc(sum(%length(&foodtype) , %length(%str(descript_)) , %length(&weight))) >
32) %then %do;
     %put '**********************************************************';
     %put "** ERROR **" ;
     %put "** The variable names of nutrient/Food (&foodtype) or survey weight is too long";
     %put "** The output files will not be suitable for the Distrib macro";
     %put "** The user need to shorten the variable names of nutrient/food or survey weight";
     %put "** Processing of the macro TRAN1 will be stopped.";
     %put '**********************************************************';
     %return;
%end;


/* have a breakdown of inputs*/
%put "**********************************************************";
%put ## Summary of Macro Inputs;
```

Supplementary data

```
%put ## Dataset is &data;
%put ## Nutrient/Food is &response, called &foodtype. in the output dataset;
%put ## Covariates (including weekend as a covariate) are &covars;
%put ## &ratioType is &ratio;
%put ## Surveyweight is &weight;
%put ## Data is sorted by ID: &subject;
%put ## Results will be saved under &outlib;
%put "********************************************************";


/**************************************************************/
/* give the zero intake as the half of the smallest non-zero value*/
/**************************************************************/
proc means data = _data min noprint;
  where &response > 0;
  var   &response;
  output out = minValue min = minValue;
run;

data _null_;
   set minValue;
     call symput("miniValue", (minValue * 0.5));
run;

%put half of min of &response is &miniValue;

data _data;
    set _data;
              if &response = 0 then &response = &miniValue;
run;

/***************************************************************************/
/* loop starts: lambda from 0.00 to 1.00 by 0.01*******************************/
/***************************************************************************/
%do i = &first_step %to &last_step;

/* boxcox transformation*/
data _data;
    set _data;
    lambda = %str(&i * &steplam);
            if _n_ = 1 then call symput("thislambda", lambda);
            if lambda = 0 then t_response = log(&response);
            else t_response = (&response**lambda - 1) / lambda;
run;

%put this lambda is &thislambda;

/* obtain the residual from y = intake and x = covariates*/
proc surveyreg data = _data;
   model t_response = &covars;
   output out = _reg
         r = resid;
                       &weight_command;
   ODS EXCLUDE html  FitStatistics ParameterEstimates DataSummary  Effects;
```

```
run;
quit;

/* get percentile 1 to 99 of the residual*/
proc univariate data = _reg noprint;
   var resid;
            &weight_command;
            output out = _pctile pctlpre = P_ pctlpts = 1 to 99 by 1;
run;

/* transpose the data to easy to understand*/
proc transpose data = _pctile out = _pctileT;
run;

/*******************************/
/* QQ plot to get the normality*/
/*******************************/

/* get the probit*/
data _pctileT;
  set _pctileT;
     prob = input(substr(_NAME_, 3), 2.) * 0.01;
     z = probit(prob);
              drop _name_ _label_;
run;

/* regress percentile on probit*/
proc reg data = _Pctilet outest = _RSquareStatistics;
  model col1 = z  / rsquare;
  ods exclude html  FitStatistics ParameterEstimates NObs ANOVA summary effect;

  run;
quit;

/* output: R-squared*/
data _RSquareStatistics;
 set _RSquareStatistics (keep = _RSQ_ );
            lambda = &thislambda;
run;

/* put all the R-squared with corresponding lambda*/
proc append data = _RSquareStatistics base = _results;
run;

proc delete data = _Pctile _Pctilet _Reg _Rsquarestatistics (gennum=all);
run;
quit;

%end;
/****************************************/
/*loop ends *****************************/
/***************************************/
```

Supplementary data

```
/**********************************************************/
/* select the optimal lambda based on the highest R-squared*/
/**********************************************************/

/* sort the dataset based on from the highest to the lowest R-squared*/
proc sort data = _results;
  by descending _RSQ_;
run;

/* select the lambda with the highest R-squared*/
data _null_;
  set _results;
    if _N_ = 1 then call symput("bestLambda", lambda);
run;

%put best lambda value &bestLambda;

/* delete _results dataset */
proc delete data = _results (gennum=all);
run;
quit;

/*****************************************/
/* generate the prediction dataset*********/
/*****************************************/

/* prepare the dataset with weekend = 0 and weekend = 1*/
data _reg;
   set _data;
     output;
            &response = .;
            &weekend = 0;
            output;
            &weekend = 1;
            output;
run;

/* transform the intake */
data _reg;
   set _reg;
     if &response = 0 then &response = &miniValue;
            if &bestlambda = 0 then t_response = log(&response);
            else t_response = (&response ** &bestlambda -1) / &bestlambda;
run;

ODS TRACE ON / label;

/* output the MSE*/
proc surveyreg data = _reg;
   model t_response = &covars;
   output out = _pred
          p = yhat;
   &weight_command;
   ods output ParameterEstimates = _params fitstatistics = _MSE;
```

```
  ods exclude html  FitStatistics ParameterEstimates  DataSummary  Effects;
run;
quit;

data _MSE;
    set _MSE;
            if Label1 = "Root MSE" then call symput("MSE", cvalue1);
run;



/* remove the oberservations with intake values, only keep the predicted intake values*/
data _pred2;
  set _pred;
     where t_response = .;
     keep &subject &weekend yhat;
run;

/* from long to wide*/
proc transpose data = _pred2 out = _pred2  prefix = x2b2_ LET;
   by &subject;
   id &weekend;
   var yhat;
run;

/* merge the dataset with weight variable */
data _weight;
  set _data;
  keep  &subject &weight;
run;

data &outlib.._pred_unc_&foodtype.;
  merge _pred2(drop = _LABEL_ _Name_) _weight;
  by &subject;
  weekend = 1;
run;

%put lambda: &bestlambda;


/*****************************************/
/* generate parameter dataset *************/
/*****************************************/
data _params;
  set _params (keep = Estimate Parameter);
run;

proc transpose data = _params out = _params2;
   id parameter;
run;

/*
* rename the variable for the distrib inputs
*/
```

Supplementary data

```
data _params;
   set _params2 (drop = _NAME_ );
            rename Intercept = A01_intercept;
run;


            %let tempcovars = tempy &covars;

            %let j=1;
            %do %while(%length(%scan(&tempcovars,&j)));
            %let curvar = %scan(&tempcovars, &j);

                        data _params;
                                    set _params;
                                    rename &curvar = A0&j._&curvar.;
                        run;


            %let j=%eval(&j+1);
            %end;

/* calculate the within/between person variance*/
data &outlib.._param_unc_&foodtype.;
  set _params;
   FreqName = "&weight";
            numvargrps = 0;
            weekendflag = 1;
   A_VAR_E = &MSE **2 * &vratio;
            A_VAR_U2 = &MSE **2 * (1- &vratio);
            A_LOGSDE = log(sqrt(A_VAR_E));
            A_LOGSDU2 = log(sqrt(A_VAR_U2));
            min_amt = &miniValue;
            A_lambda = &bestlambda;
run;

/* delete useless dataset */
proc delete data = minvalue
              _Mse
              _Params
              _Params2
              _pred
              _pred2
              _reg
              _weight(gennum=all)
              _data;
run;

quit;




%mend tran1;
```
