# Supplementary material for: A New Statistical Method for Estimating Usual Intakes of Nearly-Daily Consumed Foods and Nutrients Through Use of Only One 24-hour Dietary Recall
Source: J Nutr. 2019 Jun 7;149(9):1667–73. doi: 10.1093/jn/nxz070 (PMC6862942; doi:10.1093/jn/nxz070)
Supplement: Supplementary file 3 [file JN-2019-JN-NXZ070-S3.pdf]

Supplemental User Manual 1

TRAN1 Macro User guide

Objective: The TRAN1 macro is designed for dietary surveys that only have one dietary recall. It is used for the analysis of foods and nutrients consumed every day. The output datasets from the TRAN1 macro are used by the DISTRIB macro to estimate the distribution of usual intake.

The syntax for calling the macro is:

%macro tran1
(data=, subject =, response=, covars =, ratio = , ratioType =, weight=, weekend = , foodtype =, outlib =);

Inputs:

"data" Specifies the dataset to be used.

"subject" Specifies the variable that uniquely identifies each subject.

“response" Specifies the variable name of food or nutrient in the 24-hr recall.

"covars" Specifies a list of covariates (NOT including weekend variable). Covariates must be separated by spaces. Covariates can be either continuous variables and/or binary variables. For categorical variables, users need to recode categorical variables into multiple binary variables. This input is optional.

“Ratio” Specifies the ratio of variance component (can be in either decimal or fraction forms)

“ratioType” Specifies the type of variance ratio. The possible values are

WIVtoTotal: Within-person to total variance.

BIVtoTotal: Between-person to total variance.

WIVtoBIV: Within to Between-person variance

"weight" Specifies the survey weight. The input is optional. If weight is not specified, all the subjects will be assumed having equal weights. In general, using the NCI macro, the survey weight should be integer. In tran1, because we just use part of the NCI macro, you could have survey weight as non-integer.

"weekend" Specifies the weekend (Fri.-Sun.) indicator variable to account for a weekend effect. A value of 1 represents a Fri.-Sun. record, and a value of 0 represents a Mon.-Thurs. record.

“foodtype” Specifies a name for the analysis, used to identify the output data sets. This value can be the same as the response variable.

"**outlib**" Specifies a directory where output data sets are stored. If outlib is not specified, the output datasets will be stored under work folder in SAS. This input is optional.

data set names 'minivalue', '_Mse', '_Params', '_Params2', '_pred', '_pred2', '_reg', '_data', '_results', '_Pctile', '_Pctilet', '_Rsquarestatistics' are reserved for this macro.

Example:

```
   data exampledata;
      input studyid folate pregnant age weekendvar;
     datalines;
     1001 35.1 1 25 0
     1002 32.6 0 35 1
     1003 33.1 0 15 1
     1004 40.4 0 27 1
     1005 31.7 0 22 1
     .. see more lines ..
;


%tran1(data=exampledata,
     subject = studyid,
     response=folate,
     covars = pregnant age,
     ratio = 0.456,
     ratioType = BIVtoTotal,
     weight=,
     weekend = weekendvar,
     Foodtype = DFE,
     outlib = out);
```
